# Advances in Neurobiology and Pharmacology of GPR12

**DOI:** 10.3389/fphar.2020.00628

**Published:** 2020-05-08

**Authors:** Gonzalo Allende, Jesús Chávez-Reyes, Raquel Guerrero-Alba, Priscila Vázquez-León, Bruno A. Marichal-Cancino

**Affiliations:** Departamento de Fisiología y Farmacología, Centro de Ciencias Básicas, Universidad Autónoma de Aguascalientes, Ciudad Universitaria, Aguascalientes, Mexico

**Keywords:** GPR12, cannabinoid receptors, sphingosyl-phosphorylcholine, sphingosine-1-phosphate, cannabidiol

## Abstract

GPR12 is a G protein-coupled orphan receptor genetically related to type 1 and type 2 cannabinoid receptors (CB_1_ and CB_2_) which are ancient proteins expressed all over the body. Both cannabinoid receptors, but especially CB_1_, are involved in neurodevelopment and cognitive processes such as learning, memory, brain reward, coordination, etc. GPR12 shares with CB_1_ that both are mainly expressed into the brain. Regrettably, very little is known about physiology of GPR12. Concerning its pharmacology, GPR12 seems to be endogenously activated by the lysophospholipids sphingosine-1-phosphate (S1P) and sphingosyl-phosphorylcholine (SPC). Exogenously, GPR12 is a target for the phytocannabinoid cannabidiol (CBD). Functionally, GPR12 seems to be related to neurogenesis and neural inflammation, but its relationship with cognitive functions remains to be characterized. Although GPR12 was initially suggested to be a cannabinoid receptor, it does not meet the five criteria proposed in 2010 by the International Union of Basic and Clinical Pharmacology (IUPHAR). In this review, we analyze all the direct available information in PubMed database about expression, function, and pharmacology of this receptor in central nervous system (CNS) trying to provide a broad overview of its current and prospective neurophysiology. Moreover, in this mini-review we highlight the need to produce more relevant data about the functions of GPR12 in CNS. Hence, this work should motivate further research in this field.

## Introduction

Putative cannabinoid receptors have raised interest in the last decade as they are potential targets for developing novel pharmacologic tools, and also because many of their effects seem to involve other receptors different from type 1 and 2 receptors (CB_1_/CB_2_) (Bhattacharyya et al., 2010; Bondarenko, 2014). GPR12 is an orphan G protein-coupled receptor related phylogenetically, functionally, and pharmacologically with the cannabinoid system (see *GPR12 in the Nervous System: What Do We Know*)*?*. Many orphan receptors have been related to cannabinoids (e.g., GPR3, GPR6, GPR12, GPR18, GPR55, and GPR119 (Ryberg et al., 2007; Morales et al., 2018; Ramirez-Orozco et al., 2019). Currently, GPR55 is perhaps the most discussed protein that has been proposed as a cannabinoid receptor (Ryberg et al., 2007; Yang et al., 2016). GPR55 is expressed in the central nervous system (CNS) where it seems to be related to axonal growth control, motor control, blood flow control, anxiety, learning and memory, spatial ubication, among several other functions (Marichal-Cancino et al., 2013; Wu et al., 2013; Marichal-Cancino et al., 2014; Cherif et al., 2015; Marichal-Cancino et al., 2016; Sanchez-Fuentes et al., 2016; Marichal-Cancino et al., 2017; Marichal-Cancino et al., 2018; Ramirez-Orozco et al., 2019; Marichal-Cancino et al., 2020). GPR55 and GPR12 are targeted by cannabidiol (CBD) (Ross, 2009; Morales et al., 2018; Laun et al., 2019), a phytocannabinoid of medical interest mainly for its anticonvulsant effects (Franco and Perucca, 2019). In this mini-review, we: (i) discussed current evidence for and against GPR12 as a cannabinoid receptor (*GPR12 Biochemistry and Pharmacology*); (ii) considered the strategic expression of GPR12 in areas of the brain related to cognition and analyzed the current evidence to propose its possible physiological effects (*GPR12 in the Nervous System: What Do We Know*)*?*; and (iii) remarked the pharmacological potential that this receptor might have (*Concerns About Gpr12 as a Pharmacological Target*).

## GPR12 Biochemistry and Pharmacology

### GPR12 Biochemistry

The G protein-coupled receptor (GPCR) gene family is the major known family of mammalian membrane receptors that show a common structure with seven transmembrane domains (7TM). On the basis of their sequence homology, GPCRs were classified into five groups in line with GRAFS (i.e., Glutamate, Rhodopsin, Adhesion, Frizzled/Taste2, Secretin) classification system: glutamate receptors family (class C), rhodopsin-like receptors family (class A), adhesion receptors family (phylogenetically related to class B), frizzled/taste 2 receptors, and secretin receptors family (class B) (Schioth and Fredriksson, 2005; Alexander et al., 2017). GPR12 was cloned more than two decades ago and it belongs to the rhodopsin (class A) receptors family (Song et al., 1994; Eggerickx et al., 1995; Song et al., 1995; Marchese et al., 1999) and seems to greatly stimulate adenylate cyclase in the absence of ligand-receptor interaction; thus, it is considered a “constitutively active receptor”. In other words, GPR12 has the property of exhibiting a basal G protein-coupling activity, since it upregulates the adenylate cyclase signaling cascade without any activating ligand (Eggerickx et al., 1995; Uhlenbrock et al., 2002; Martin et al., 2015). GPR12 seems to couple to both Gαs and Gαi proteins since it stimulates baseline cAMP-dependent signaling by activating Gαs proteins, and also inhibits forskolin activated signaling through Gαi interaction (Uhlenbrock et al., 2002; Martin et al., 2015). Tanaka et al. (2007) showed evidence that GPR12 exerts the same intracellular pathway on neurons by upregulating cAMP pathway and consequently activating protein kinase A (PKA). Overexpression of GPR12 in a non-neuronal cell line, such as HEK293, significantly increased basal levels of cAMP (Tanaka et al., 2007). GPR12 also mediates the calcium release through a Gαi signaling cascade that partly involves the activation of intracellular sphingosine kinase (Uhlenbrock et al., 2002). Interestingly, GPR12 activity seems to depend on external lipids, because the culture of HEK293 cells overexpressing GPR12 in a lipid-free medium resulted in a decrease in basal cAMP production, compared to cells grown in a standard medium (Uhlenbrock et al., 2002).

GPR12 shares a high amino acid sequence identity with GPR3 and GPR6 ranging from 60–68% in TM regions, 45% with both receptors of the lysophospholipid LPA1 and sphingosine-1-phosphate, and with the cannabinoid receptors about a 35% identity in the TM regions (Lee et al., 2001; Hauser et al., 2017; Ye et al., 2019).

Structurally, GPR12 is composed of 334 amino acids (Song et al., 1995) and was initially cloned in 1991 from the rat pituitary gland and originally named R334 (Eidne et al., 1991). GPR12 was later cloned from mouse in 1993, and the authors then designated it as GPCR21 (Saeki et al., 1993). Thereafter, GPR12 was cloned from a human DNA library along with two other closely related receptors: GPR3 and GPR6 (Song et al., 1995). Recently, Ye et al. (2019) placed GPR12 protein (besides GPR3, GPR6, and others) as a possible cannabinoid receptor candidate.

### GPR12 Pharmacology

For decades, lysophospholipids have been described as extracellular ligands for GPCRs involved in numerous cellular processes, such as apoptosis, proliferation, differentiation, and motility (Van Brocklyn et al., 1998; Ancellin and Hla, 1999; Fukushima et al., 2000; Dyatlovitskaya, 2007). S1P (Uhlenbrock et al., 2002), SPC (Ignatov et al., 2003), and tyrosol (Lin et al., 2008) were reported as high-affinity ligands for GPR12. Activation of GPR12 by S1P promoted mobilization of intracellular Ca^2+^ (Uhlenbrock et al., 2002). Ignatov et al. (2003) carried out the heterologous expression of GPR12 in frog oocytes and reported that both S1P and SPC activate the receptor. In addition, Bresnick et al. (2003) confirmed a functional association of GPR12 with Gαs protein using a HEK293 cell line overexpressing the receptor. Uhlenbrock et al. (2002) found that GPR12 mediates the calcium release through the Gαi signaling cascade partly *via* sphingosine kinase activity. They also found that constitutive activation of adenylate cyclase and mobilization of Ca^2+^ can be modulated by the sphingophospholipid S1P (Uhlenbrock et al., 2002).

The dual-coupling is a previously described phenomenon supporting the idea that GPCR receptors could activate more than one intracellular pathway (Herrlich et al., 1996; Daaka et al., 1997; Hermans, 2003). In this regard, several mechanisms have been proposed to explain the promiscuity of some GPCR receptors, including the involvement of different receptor domains to interact with several Gα subunits, as well as the participation of splice variants of the receptors (Hermans, 2003). Pharmacological approaches could help solve this problem, however, to date, there are no reports describing specific agonists and antagonists for GPR12, which makes it difficult to identify the signal transductions associated with GRP12.

GPR12 transfected cells in charcoal-stripped serum significantly decreased constitutive Gαs signaling and this reduction could be partly recovered in the presence of S1P, suggesting agonistic effects on adenylate cyclase stimulation (Uhlenbrock et al., 2002). In contrast with those results, Hinckley et al. (2005) only observed a very small significant increase in cAMP accumulation induced by S1P in COS7 cells transfected with mouse GPR12 cDNA. Likewise, later studies could not confirm that S1P is a real endogenous ligand for GPR12 (Yin et al., 2009), since it was reported that GPR12 showed no activity in β-arrestin PathHunterTM assays for the ligand S1P (Yin et al., 2009; Southern et al., 2013).

Ignatov et al. (2003) demonstrated that lysophospholipid SPC is a high affinity ligand for GPR12, with a hundred times higher potency and efficacy if compared to S1P. They showed that in CHO cells and Xenopus oocytes transfected with GPR12, SPC induced a significant increase in Ca^2+^ intracellular levels and a strong GIRK-mediated inward current, respectively (GIRK = G protein-gated inwardly rectifying K^+^ channel). These effects of SPC were blocked using pertussis toxin, suggesting that GPR12 is coupled to an inhibitory G-protein. They also reported that SPC was found to increase synaptic contacts in cultures of mouse embryonal cerebral cortical neurons, as well as increased cell proliferation and cell clustering in mouse hippocampal HT22 cells (38). Later, a Park et al. (2016) study showed that GPR12 overexpression stimulated keratin 8 (K8) phosphorylation and reorganization even in the absence of ligand SPC.

SPC is a sphingolipid present at low concentrations in blood plasma, it is originated from the hydrolysis of sphingomyelin. In recent years, different studies have suggested SPC as an intracellular second messenger or an extracellular ligand of unidentified G protein-coupled receptors (Nixon et al., 2008; Yue et al., 2015). Considering the experiments developed by Ignatov et al. (2003), SPC may be an endogenous agonist to GPR12 since the EC50 reported, using heterologous expression of GPR12, was 32 nM (Ignatov et al., 2003). The above value may be physiological reached as the serum concentration of SPC is around 50 nM in plasma and 130 nM in serum (Liliom et al., 2001). Indeed, experiments achieved to demonstrate the activation of GPR12 by SPC showed results at nanomolar (Ignatov et al., 2003) up to micromolar concentrations (Park et al., 2016). Other experiments that involved biological effects of SPC *via* unidentified mechanisms on several cellular models are induced at concentrations from 0.5 to 30 μM (Törnquist et al., 1995; Van Koppen et al., 1996; Himmel et al., 1998; Beil et al., 2003).

An potential phenomenon to consider when studying the biological effects of higher concentrations of lipids as SPC is their detergent-like actions (Contreras et al., 2006). Nevertheless, as the critical micelle concentration (CMC) reported to SPC is 158 μM (measured in a solution pH 7.4 at 25°C) (Li et al., 2004), the results obtained using concentrations ranged nanomolar to 30 μM (as the previously described) could not be explained in terms of a detergent-like action of SPC. Supporting the above idea, Contreras et al. (2006) reported that CMC for Sphingosine was 18 μM; but, interestingly, they concluded that there was not a detergent action at this concentration when probed the Sphingosine on liposomes containing fluorescent molecules (Contreras et al., 2006). Nevertheless, they reported that some of the physiological effects of high doses of sphingosine may be explained for a breakdown in the membrane permeability (Contreras et al., 2006).

In any case, more experimental evidence (e.g., to explore that tissue localization of GPR12 match with local production of SPC at a nM range, among others) is required to confirm or discard that SPC acts as an endogenous agonist to GPR12.

On the other hand, Du et al. (2008) reported that five steroid compounds isolated from cultures of the volcanic ash-derived fungus *Penicillium citrinum* HGY1–5, induced the cAMP production in GPR12-transfected CHO cells. The authors found that the induction of cAMP generation depends on the receptor, since cAMP was not detected in wild-type CHO cells. However, they mentioned that more experiments should be performed to determine if these compounds could be specific agonists for GPR12 [48].

Park et al. (2016) studied the effects of two synthetic drugs: FTY20 and FTY20P (potent S1P1,3,4,5 receptor agonists) and reported that both drugs suppressed the GPR12-induced phosphorylation and reorganization of K8 by restoring the expression of protein phosphatase 2A (PP2A) *via* GPR12 (Park et al., 2016). Interestingly, CBD is an inverse agonist for cAMP accumulation acting on GPR12 (Brown et al., 2017). This group used different endocannabinoids and phytocannabinoids and found that CBD was the best phytocannabinoid to reduce significantly GPR12 mediated cAMP accumulation with the 10 and 100 µM concentrations tested in HEK293 cells expressing GPR12. In addition, also the endocannabinoids 2-arachindonylglycerol and virodhamine significantly reduced cAMP accumulation with 100 µM, the highest concentration tested (Brown et al., 2017). They also carried out experiments using cholera toxin (CTX) and pertussis toxin (PTX) to evaluate the involvement of G proteins in GPR12 mediated cAMP accumulation. When CTX was used to block Gαs signaling there was a suppression of CBD inverse agonist, which was not observed when PTX was used to block Gαi signaling, indicating that Gαs but not Gαi protein is involved in the inverse agonism of CBD on GPR12 mediated cAMP accumulation. Therefore, the authors concluded that, at micromolar concentrations, CBD acts as an inverse agonist on GPR12, defining GPR12 as a novel molecular target for CBD (Brown et al., 2017). Finally, the authors performed structure-activity relationship studies of CBD as an inverse agonist of GPR12, and their data suggests that both: the length of the pentyl side chain and the two free hydroxyl groups located on the benzene ring are critical for CBD to exhibit its inverse agonistic activity on GPR12 mediated cAMP accumulation (Brown et al., 2017). Shortly before the release of that report, the same group indicated that CBD also acts as an inverse agonist on both GPR3 and GPR6 orphan receptors (Laun and Song, 2017). Laun et al. (2019) also reported GPR3, GPR6, and GPR12 as molecular targets for CBD. These three receptors belong to the rhodopsin (class A) receptors family (Alexander et al., 2017), and they show high similarity (60%) between them as regards their amino acid sequences (Song et al., 1995). Since CBD shows low affinity at these orphan GPR receptors presenting IC50 at micromolar concentration, it is a promiscuous compound with activity at multiples targets (Mcpartland et al., 2015), and there is not pharmacological studies demonstrating the direct interaction between CBD and GPR12. The possibility that CBD is exerting its effects through an indirect mechanism of action is not ruled out, for this reason, additional research is required to determine that CBD is a ligand of GPR12.

Potentially, S1P and/or SPC could be endogenous ligands of GPR12; however, the sequence of GPR12 is very different from other putative SPC receptors, such as OGR1 and GPR4 (Im, 2004). In addition, despite the experiments described above, where S1P or SPC activated the GPR12 receptor to produce an intracellular response, conclusive experiments that demonstrate a direct interaction between an endogenous ligand and GPR12 (eg, binding assays) are lacking. More evidence is still required to consistently demonstrate that SPC is the endogenous ligand of GPR12. Therefore, GPR12 is today still considered an orphan receptor, since no endogenous ligand has so far been confirmed.

### Does GPR12 Meet the Criteria to be Classified as a Cannabinoid Receptor?

GPR12 has a sequence similarity at the transmembrane level over 40% homology with the cannabinoid receptor CB_1_ and CB_2_ (Morales et al., 2018) and some sphingolipids have been proposed as the putative endogenous ligand for GPR12 (Ignatov et al., 2003) [25]. Hence, it is understandable the speculation about its possible association with the cannabinoid receptors. However, in 2010, criteria for classifying cannabinoid receptors were suggested by a special committee of the International Union of Basic and Clinical Pharmacology (IUPHAR; **Table 1**) (Pertwee et al., 2010); GPR12 only partially meets these criteria. Therefore, it couldn’t be classified as a cannabinoid receptor. To date, only two proteins are officially classified by IUPHAR as cannabinoid receptors (Console-Bram et al., 2012): (i) CB_1_ that was described and cloned in 1988 and 1990, respectively (Devane et al., 1988; Matsuda et al., 1990); and (ii) CB_2_ that was described in macrophages in 1993 (Munro et al., 1993).

**Table 1:** Proposed criteria for classifying cannabinoid receptors according to IUPHAR in 2010 (Pertwee et al., 2010).

Criteria	Current IUPHAR-proposed criteria for classifying cannabinoid receptors	GPR12 as a potential cannabinoid receptor	References
**Criteria 1**	It should be activated at its orthosteric site and with significant potency by an established CB_1_/CB_2_ receptor ligand.	No data are available about this criterion.	N/A
**Criteria 2**	It should be activated by at least one established endogenous CB_1_/CB_2_ receptor agonist at “physiologically relevant” concentrations.	It does not meet the criterion. GPR12 seems to be activated by Sphingosine-1-phosphate that also interact with CB_1_ receptors. But, there is not confirmation of any established endogenous CB_1_/CB_2_ receptor agonist that interacts at “physiologically relevant” concentrations with GPR12.	(Uhlenbrock et al., 2002; Ignatov et al., 2003; Paugh et al., 2006)
**Criteria 3**	If it is a GPCR, it should display significant amino acid sequence similarity with the CB_1_ or the CB_2_ receptor, which are members of the α group of rhodopsin-type GPCRs.	It meets this criterion, as GPR12 shares amino acid sequence similarity with both CB_1_ and CB_2_ (around 40%) and belongs to the α group of rhodopsin-type GPCRs. It is important to highlight that its maximal similitude is with GPR3 and GPR6 (~56–59%).	(Lee et al., 2001; Fredriksson et al., 2003; Morales et al., 2017)
**Criteria 4**	It should not be a “well established” non-CB_1_/CB_2_ receptor or channel, especially if there is already strong evidence that 1) this is endogenously activated by a non-CB_1_/CB_2_ receptor ligand with appropriate potency and relative intrinsic activity and 2) this is not endogenously activated by any endocannabinoid with appropriate potency and relative intrinsic activity.	It meets the criterion as GPR12 is not a “well established” non-CB_1_/CB_2_ receptor.	(Pertwee et al., 2010)
**Criteria 5**	It should be expressed by mammalian cells that are known to be exposed to concentrations of endogenously released endocannabinoid molecules capable of eliciting a response.	It partially meets the criterion as GPR12 expression in CNS is quite similar to that of CB_1_-high expression brain areas (e.g., striatum). However, there is no evidence that endocannabinoid molecules can elicit a response *via* GPR12.	(Ignatov et al., 2003)

Initially, the cognitive and behavioral effects induced by cannabinoids (which include phytocannabinoids, endocannabinoids, and synthetic cannabinoids) were attributed to CB_1_ receptors, while non-nervous and mainly immune peripheral effects were related to CB_2_ receptors. That previous notion was overcome by the evidence, since it is currently known that both receptors have medical implications in nervous and non-nervous cells, immune and non-immune cells, and in virtually all cell types throughout the human body (Buckley et al., 1997; Svizenska et al., 2008; Schrot and Hubbard, 2016; Lu and Anderson, 2017).

CB_1_ and CB_2_ receptors are related to most of the effects induced by exogenous cannabinoids; but some effects may include putative cannabinoid receptors and other targets, such as cationic channels or nuclear receptors. Lamentably, many of the conclusions about cannabinoids in the last decades have been achieved from the assumption of endocannabinoids and synthetic cannabinoids as mainly CB_1_/CB_2_ agonists and the use of CB_1_/CB_2_ antagonists designed without the corresponding binding assays against all non CB_1_/CB_2_ potential targets currently known. Thus, we consider that the simplest use of the classic CB_1_/CB_2_ antagonists (e.g., rimonabant and AM630) for studying the pharmacological profile of the effects of cannabinoids is not enough to conclude any CB_1_/CB_2_ participation. Thus, we propound that the follow profiles must be absent in the effects of cannabinoids to suggest a main role for CB_1_/CB_2_ receptors: (i) the lack of a dose-dependent behavior; (ii) opposite effects at higher doses; and (iii) persistent effects of cannabinoids in mutant animals that lack CB_1_ and/or CB_2_ receptors or gene silencing techniques. Any of the previous profiles may suggest the participation of additional cannabinoid receptors not classified yet.

The classical criteria for classifying pharmacological receptors have involved three main properties: (i) biochemical; (ii) pharmacological; and (iii) molecular characteristics. The criteria according to IUPHAR (Pertwee et al., 2010) for classifying cannabinoid receptors are shown in **Table 1**.

According to the information so far available, GPR12 only partially meets some of the criteria for being classified as a cannabinoid receptor and, therefore, it must continue to be considered as an orphan receptor until new evidence proves otherwise. Moreover, we think it is time to ask whether it is appropriate to keep the cannabinoid nomenclature considering them as a specific group, or if it would be better to reclassify some of the lipid receptor groups in more general families (e.g., a great family of receptors for N-acylethanolamides). So far, the cannabinoid system is ubiquitous and related to multiple cannabinoids, non-cannabinoid and orphan receptors throughout the organism, including GPR12.

## GPR12 in the Nervous System: What Do We Know?

### GPR12 Expression and Function in the Mammal Brain

In the CNS, GPR12 has been mainly detected in structures closely related to cognitive processes, including learning, memory, and the brain reward system, such as the cerebral cortex, the hippocampus, and the striatum (Saeki et al., 1993). Interestingly, behavior in mutant mice lacking GPR12 seems to be normal (Frank et al., 2012). Unfortunately, experiments performed to analyze the physiological role of GPR12 in cognitive processes are scarce.

Indirect findings allow to speculate that this receptor could be playing a role in brain plasticity as it is involved in promoting neurite outgrowth (Tanaka et al., 2007) (see *GPR12 Involvement in Neural Development* ***)***. However, its functional impact on behavior has not yet been characterized. Currently, there are two indirect clues to follow in order to elucidate the cognitive actions of GPR12: (i) behavior of mutant mice lacking the GPR12 gene; and (ii) analysis of the cognitive effects of CBD (which interact with other receptors such as GPR55 and CB_1_). GPR12 mRNA was also detected in rat pituitary, piriform cortex and lateral septal nuclei (Eidne et al., 1991), as well as in mouse frontal cortex, thalamus, hypothalamus, and olfactory bulb (Saeki et al., 1993). But again, its physiology remains obscure.

**Figure 1:**
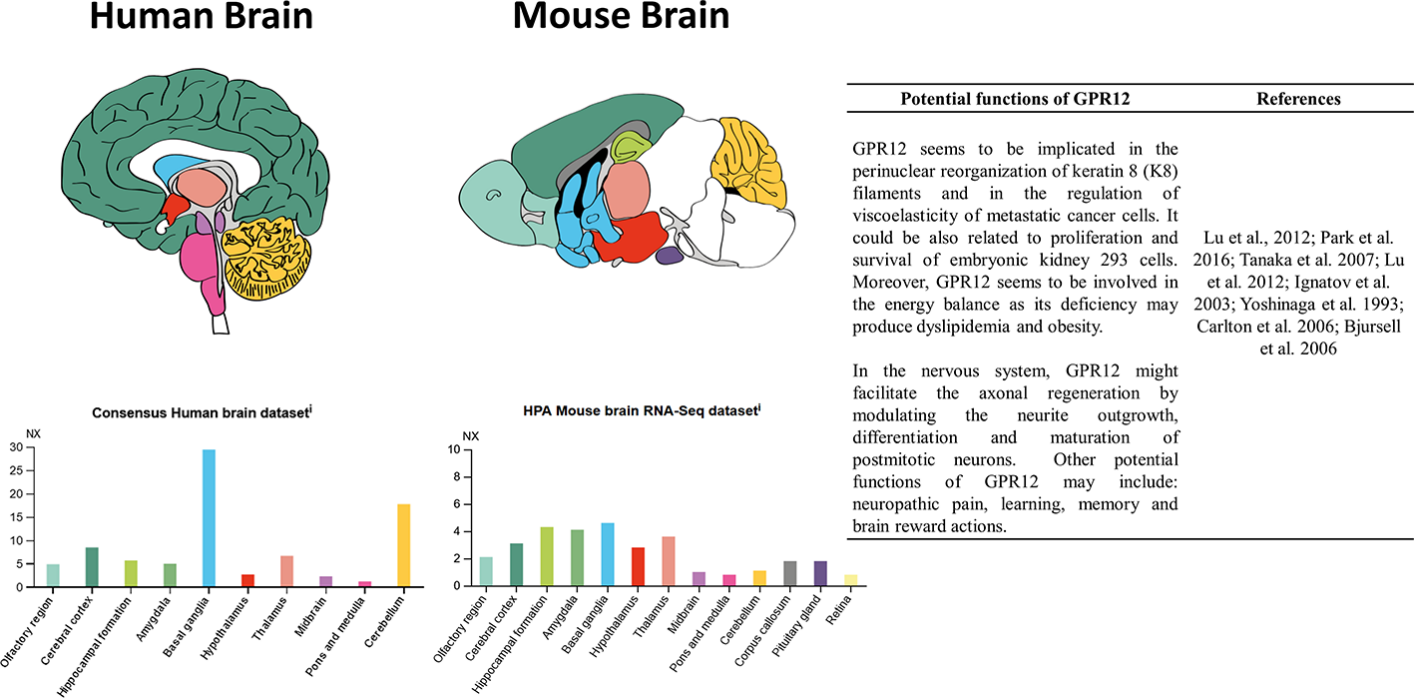
RNA detection of GPR12 in main human and mouse brain areas and some of its potential roles. Images taken from “The Human Protein Atlas®” available at: https://www.proteinatlas.org/ENSG00000132975-GPR12/brain.

In human CNS, GPR12 is mainly expressed in the basal ganglia, the cerebellum, and the cerebral cortex, in a lesser extent in the thalamus, the hippocampus, the olfactory region, and the amygdalae, and scarcely expressed in the hypothalamus, the midbrain, and the corpus callosum (**Figure 1**; https://www.proteinatlas.org/).

GPR12 has been associated with neuropathic pain. In fact, the company “Paradigm Therapeutics Ltd” patented the use of SPC and other GPR12 ligands to influence the neuronal and limbic system in pain treatment (Carlton et al., 2006). They reported that GPR12 knockout mice were more sensitive than wild type mice to heat-induced pain in the tail-flick and hot plate test. In the future, many clues are open to investigating the role of GPR12 in pain pathways, and also the functions of other putative cannabinoid receptors, such as GPR18 and GPR55 (Guerrero-Alba et al., 2018).

### GPR12 Involvement in Neural Development

GPR12 mRNA expression during embryonal development was analyzed through *in situ* hybridization. In the embryonal mouse brain, a strong expression of GPR12 was detected in the cortical plate, the piriform cortex, and the hippocampus (Ignatov et al., 2003). Evident signals were also found in the dorsomedial and arched nuclei, and weaker ones were detected in the mamillary body (Ignatov et al., 2003). In addition, GPR12 expression was upregulated at neuronal differentiation regions, whereas it was absent in neuroblast proliferation areas, such as the ventricular zone. Ignatov et al. (2003) provided evidence that embryonic cerebral cortical neurons treated with SPC, a high-affinity ligand for GPR12, exhibited an increase in the number of synaptic contacts as well as in synaptophysin expression. Furthermore, SPC stimulated proliferation and cell clustering in mouse hippocampal HT22 cells, suggesting that SPC, possibly interacting with GPR12, has positive effects on differentiation and maturation of postmitotic neurons (Ignatov et al., 2003). An elegant study carried out by Saeki and coworkers indicated that up-regulated GPR12 expression could facilitate axonal regeneration in rat cerebellar granule neurons through the GPR12 constitutive action, which increases intraneuronal cAMP promoting neurite outgrowth and blocking myelin inhibition in rat primary neurons (Tanaka et al., 2007). Similar results were reported by Lu et al. (2012a) using rat pheochromocytoma PC12 cells, who showed that GPR12 overexpression induced PC12 cells differentiation into neuron-like cells with enlarged cell sizes and neuritogenesis. The authors suggested that this effect could be determined by the activation of Erk1/2 signaling pathway and the increased expression of several neurite outgrowth-related genes (Lu et al., 2012a). Almost simultaneously, the same group demonstrated that heterologous GPR12 expression promoted proliferation and survival in HEK293 cells, probably through the activation of an extracellular signal-regulated protein kinase which would increase Erk1/2 and B-cell lymphoma/leukemia-2 expression in HEK293 cells overexpressing human GPR12 (Lu et al., 2012b). In addition, Neuro2a cells transfected with GPR12 exogenous gene showed a prominent neurite outgrowth, and this effect was abrogated using a PKA pharmacological antagonist, KT5720. These experiments suggested that GPR12 regulates neurite outgrowth through the cAMP-PKA pathway (Tanaka et al., 2007). Furthermore, it was observed that GPR12 is largely expressed in microglia, since it was co-expressed with the microglia marker: ionized calcium-binding adapter molecule 1 (Iba1), and that it was upregulated after exposition to cuprizone, a demyelinating toxin (Bedard et al., 2007). The latter suggests that GPR12 could have a role during inflammation/regeneration events in the CNS.

### GPR12 Non-Neural Expression in the Human Body

So far, GPR12 has been scarcely detected in human peripheral tissues. Thus, RNA expression analysis shows that GPR12 is acceptably expressed in the salivary glands, moderately expressed in the breast and skin and, to a lesser extent, in the fallopian tube and granulocytes (https://www.proteinatlas.org/). Previously, GPR12 had also been found in human umbilical vein endothelial cells, where fluid shear stress increases the expression of this receptor (Uhlenbrock et al., 2002).

One of the most studied GPR12 mediated effects is the meiotic arrest in oocytes. Hinckley et al. (2005) reported that GPR12 seems to stimulate Gαs activity in a ligand-independent manner (constitutive activity), at least in their reconstituted systems in mouse and rat oocytes, and the occupancy of this receptor by the ligand SPC resulted in a consistent and significant delay in oocyte maturation (Hinckley et al., 2005). These results could be logically explained assuming that SPC might be acting as an inverse agonist, counteracting the GPR12 constitutive activity responsible for the cAMP increase. Di Luigi et al. (2008) also indicated that meiotic arrest in human oocytes is maintained by a Gαs signaling pathway, but they also suggested that such event is mediated only by GPR3 and not by GPR12, as the mRNA of this last receptor was not detected using PCR in human oocytes (Diluigi et al., 2008). Subsequently, another research group mentioned that it seems possible that GPR6 and GPR12 could be relevant proteins in the maintenance of meiotic arrest in Xenopus laevis oocytes (Rios-Cardona et al., 2008).

Conversely, Vaccari et al. (2008), working with mouse oocytes, indicated that meiotic arrest is mediated by GPR3, suggesting that cAMP accumulation derived from the receptor activity is important to maintain the meiotic arrest in the mouse oocyte (Vaccari et al., 2008). It has also been proposed that GPR12 expression is important for the cAMP levels required for meiotic arrest [42].

The targeted disruption of adenylate cyclase-activating polypeptide gene has proved that this peptide has a role in lipid and carbohydrates metabolism, as well as in the sympathetic response to insulin stress and body temperature (Gray et al., 2002). In line with this finding of adenylate cyclase-activating polypeptide gene disruption, Bjursell et al. (2006), working with GPR12 knockout mice, reported that GPR12 is important for energy balance, since GPR12 deficiency resulted in dyslipidemia and obesity in mice. The authors proposed that genetic disruption of GPR12 provokes changes in both lipid and carbohydrate metabolism, probably due to the ability of this receptor to regulate the energy expenditure (Bjursell et al., 2006). However, later results obtained by the same group indicated that metabolic parameters are not significantly affected in GPR12 mutant mice (Frank et al., 2012).

It has also been suggested that GPR12 may be implicated in the perinuclear reorganization of K8 filaments and in the regulation of the viscoelasticity of metastatic cancer cells, leading to an enhanced migration (Park et al., 2016). Sphingosyl-phosphorylcholine induced K8 phosphorylation and reorganization in PANC-1 human pancreatic carcinoma cells, probably through the interaction of this ligand with GPR12. This effect could be explained by the SPC-induced reduction of PP2A expression, which is an upstream regulator of Jun N-terminal kinase (JNK) and extracellular receptor kinase (ERK). Moreover, it was demonstrated that GPR12 overexpression stimulated keratin phosphorylation and reorganization even without SPC, confirming its constitutive activity in the absence of an agonist. It was also reported that fingolimod (FTY720), a sphingosine-related molecule with immunomodulatory function, suppressed SPC-induced phosphorylation and reorganization, with the subsequent cell migration by restoring the expression of PP2A *via* GPR12, possibly because FTY720 competes with SPC for this receptor. These findings could be useful for the future design of compounds that may modulate the cell migration of metastatic cancer cells (Park et al., 2016).

Recently, Ge et al. (2018) proposed some potential roles for SPC in the cardiovascular system through modulation of myocytes, vein endothelial, and vascular smooth muscle functions. The authors assume that, as SPC is a constituent of lipoproteins and its liberation into the blood is promoted by the activation of platelets, it could thus trigger certain effects to modulate some heart and vessel pathological processes. The authors indicated that some studies suggest that SPC acts as a first messenger through G protein-coupled receptors, like S1P1–5 or GPR12, or through membrane lipid rafts, or as a second messenger mediating intracellular Ca^2+^ release (Ge et al., 2018).

Some years ago, the phytocannabinoid CBD was reported to be an inverse agonist for GPR12 (Brown et al., 2017). CBD is an active constituent of cannabis, and unlike tetrahydrocannabinol (THC), the most psychoactive component of the plant *Cannabis sativa*, CBD is a non-psychotropic component (Izzo et al., 2009). CBD has been pointed out as a ligand capable to interact with many targets, including enzymes and receptors, and has also been proposed as a novel agent for numerous therapeutic applications, among others as a potential anticancer drug (Massi et al., 2013; Pisanti et al., 2017).

Brown et al. (2017) demonstrated that CBD acts on GPR12 as an inverse agonist to inhibit the cAMP accumulation stimulated by this constitutively active receptor, and the use of cholera toxin to block Gαs signaling showed that Gαs is involved in the CBD inverse agonistic activity on GPR12-mediated cAMP accumulation (Brown et al., 2017).

## Concerns About GPR12 as a Pharmacological Target

Although there are currently no commercially available pharmacological tools to stimulate or block GPR12, some effects can be assumed in line with the physiological processes in which this receptor is involved. According to the experiments performed to date, the GPR12 blockade could interrupt the development of neuronal tissue. Ignatov and his colleagues obtained data that suggest the positive role of GPR12 in the differentiation and maturation of postmitotic neurons (Ignatov et al., 2003); additionally, rat cerebellar granular neurons overexpressing GPR12 showed extensive neurite growth, around eight times faster than control cells (Tanaka et al., 2007). In fact, GPR12 overexpression in PC12 cells induced differentiation in neuron-like cells, since neurite outgrowth was observed in transfected cells (Lu et al., 2012a). These data allow us to assume that blocking GPR12 could cause a blockage of neurite growth. However, and despite the relevant functions of GPR12 in neuronal homeostasis, it seems difficult to assess physiological alterations after blocking GPR12, considering that GPR3 and/or GPR6 coexist in tissues with GPR12 and they also could perform the functions of GPR12 and vice versa. Supporting the above mentioned, Tanaka and his colleagues carried out a knockdown of GPR3 in neurons of rat cerebellar granules that produced a significant inhibition of neurite growth and then observed that this inhibitory effect was reversed by the co-transfection of GPR12 (Tanaka et al., 2007). Thus, they concluded that GPR3 and GPR12 act redundantly at least in this phenomenon. Namely, GPR3 knockout mice showed apparent minor neurological alterations, such as behavioral alterations (Valverde et al., 2009), modified sensitivity to thermal non-noxious and noxious stimuli (Ruiz-Medina et al., 2011), and alterations in some cognitive functions (Tourino et al., 2012). Indeed, in other experiments using GPR12 knockout mice, compared to wild-type controls, showed no differences in any parameter in the behavioral tests related to emotionality (Frank et al., 2012). To date, many less significant alterations (for example, inhibition of neuronal tissue development or neuronal morphological changes) have been reported, probably due to the redundancy described above. In contrast, neural alterations derived from the absence of GPR12 in mice resulted in a phenotype of obesity and dyslipidemia, which suggests a possible role of this receptor in energy expenditure and energy homeostasis (Bjursell et al., 2006).

### Conclusions

GPR12 does not meet the current criteria to be classified as a cannabinoid receptor. These criteria and the alternative of considering N-acyl-ethanolamines as a broader family of receptors should be reviewed to allow better integration of the information gathered on the subject. GPR12 is an interesting receptor mainly expressed in the CNS, but evidence on its physiological role is limited to pain issues and neurodevelopment. It is possible that this receptor participates in cognitive and behavioral events such as learning, memory, and food ingestion. Efforts should be focused on confirming those potential functions, or finding other different functions effectively mediated through GPR12.

## Author Contributions

GA-A developed the manuscript, corrected the style, reviewed and edited the manuscript and discussed the central ideas of it. JC-R developed the manuscript and discussed the central ideas of it. RG-A developed the manuscript and discussed the central ideas of it. PL designed the graph, reviewed and edited the manuscript. BM-C developed the manuscript, proposed the central idea of it, designed the graph and the table, reviewed and edited the manuscript and acquired funding.

## Funding

This work was supported by "Decanato del Centro de Ciencias Básicas" from Autonomous University of Aguascalientes.

## Conflict of Interest

The authors declare that the research was conducted in the absence of any commercial or financial relationships that could be construed as a potential conflict of interest.
